# Risk gene-set and pathways in 22q11.2 deletion-related schizophrenia: a genealogical molecular approach

**DOI:** 10.1038/s41398-018-0354-9

**Published:** 2019-01-17

**Authors:** Elena Michaelovsky, Miri Carmel, Amos Frisch, Mali Salmon-Divon, Metsada Pasmanik-Chor, Abraham Weizman, Doron Gothelf

**Affiliations:** 1 Sackler Faculty of Medicine, Tel Aviv University, Tel Aviv, Israel; 2 Felsenstein Medical Research Center, Petah Tikva, Israel; 3 Department of Molecular Biology, Ariel University, Ariel, Israel; 4 Bioinformatics Unit, G.S. Wise Faculty of Life Science, Tel Aviv University, Tel Aviv, Israel; 5 Geha Mental Health Center, Petah Tikva, Israel; 6 Sagol School of Neuroscience, Tel Aviv University, Tel Aviv, Israel; 7 The Behavioral Neurogenetics Center, Sheba Medical Center, Tel Hashomer, Israel

## Abstract

The 22q11.2 deletion is a strong, but insufficient, “first hit” genetic risk factor for schizophrenia (SZ). We attempted to identify “second hits” from the entire genome in a unique multiplex 22q11.2 deletion syndrome (DS) family. Bioinformatic analysis of whole-exome sequencing and comparative-genomic hybridization array identified de novo and inherited, rare and damaging variants, including copy number variations, outside the 22q11.2 region. A specific 22q11.2-haplotype was associated with psychosis. The interaction of the identified “second hits” with the 22q11.2 haploinsufficiency may affect neurodevelopmental processes, including neuron projection, cytoskeleton activity, and histone modification in 22q11.2DS-ralated psychosis. A larger load of variants, involved in neurodevelopment, in combination with additional molecular events that affect sensory perception, olfactory transduction and G-protein-coupled receptor signaling may account for the development of 22q11.2DS-related SZ. Comprehensive analysis of multiplex families is a promising approach to the elucidation of the molecular pathophysiology of 22q11.2DS-related SZ with potential relevance to treatment.

## Introduction

Schizophrenia (SZ) is a disabling mental disorder affecting 1% of the general population with a strong genetic component. Case–control studies of thousands participants have identified many molecular variants carrying genetic risk for SZ^1–3^. The highest known genetic risk for SZ is the 22q11.2 microdeletion^1^. Individuals carrying a hemizygous 22q11.2 deletion, “first hit”, have ~40% chance of developing SZ-spectrum disorders in adults^4^.

Since not all individuals who carry the 22q11.2 deletion develop SZ, it is plausible that genetic variants outside the 22q11.2 region (“second hits”) contribute to the risk to develop SZ^5^. In an attempt to identify genetic risk factors contributing to SZ development, we focused on a large multiplex 22q11.2DS family. Studying multiplex families is a promising approach since the risk variants in the SZ affected individuals are expected to be enriched compared to the non-affected family members. A genealogical analysis, in contrast to case–control study, eliminates the need for population stratification. Moreover, it is a straightforward and less expensive strategy to analyze the association between clinical and genetic data in a particular family under a long-lasting close clinical follow-up.

**Fig. 1:**
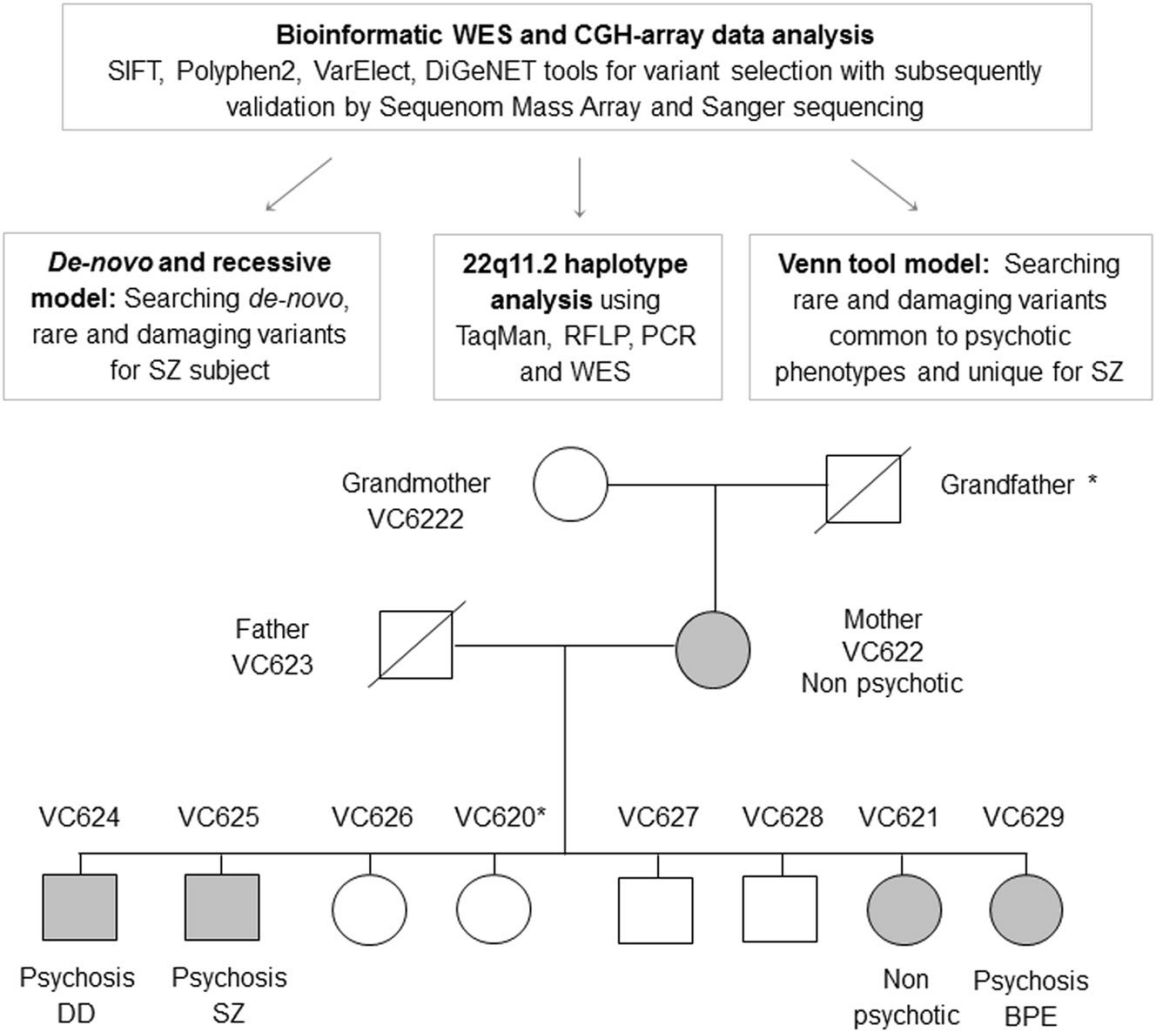
**The analysis process designed for identifying molecular variants that may contribute to SZ-spectrum disorders in a multiplex 22q11.2DS family.** Filled symbols indicate 22q11.2DS affected person with the same 22q11.2 deletion (CGH-array: chr22:18,844,632-21,703,145, GRCh37). *No DNA was available for these family members. BPE brief psychotic episode, CGH comparative-genomic hybridization, CNV copy number variation, DD delusional disorder, MLPA multiplex ligation probe amplification, SZ schizophrenia

We took the advantage of a unique multiplex 22q11.2DS family with high load of psychosis expression (Fig. 1) and attempted to identify “second hits” that may contribute to psychosis and SZ developing, using bioinformatic analysis of whole-exome sequencing (WES) and comparative-genomic hybridization (CGH) array.

## Materials and methods

### Subjects

The multigeneration Jewish family of Moroccan origin, consisted of a grandmother (mother side), parents and 8 children, referred to the Behavioral Neurogenetics Center with suspected 22q11.2DS in one of the children (VC621). Genetic assessment has demonstrated that VC621, her mother and three of her seven siblings are affected with 22q11.2DS (Fig. 1). All family members with 22q11.2DS had psychiatric manifestations with varying degrees of severity, including specific and social anxiety disorder, generalized anxiety disorder (GAD), attention deficit/ hyperactivity disorder (ADHD), and SZ-spectrum disorders, including SZ, delusional disorder (DD), and history of brief psychotic episode (BPE) (Supplementary Table 1).

The SZ-VC625 patient was hospitalized at the age of 25 years due to an acute psychotic episode that was triggered by use of 3,4-methylenedioxymethamphetamine (MDMA) and heroin. He was diagnosed with paranoid SZ and substance use disorder. Since then he suffers from chronic psychotic symptoms and marked negative symptoms with poor response to antipsychotics and he is currently living in a sheltered home. VC624 was diagnosed as suffering from a DD at the age 43. VC629 was diagnosed at the age 12 with ADHD, GAD, specific and social anxiety disorders and experienced a BPE at age 14. Unfortunately, no DNA was available for the sibling VC620 (Fig. 1) and therefore she was not included in the molecular analyses.

### Psychiatric and cognitive assessments

The participants were interviewed by experienced clinicians using the Structured Clinical Interview for Axis I DSM-IV Disorders (SCID)^6^. The ADHD module from the Schedule for Affective Disorders and Schizophrenia for School-Aged Children, Present and Lifetime^7^ was added to the SCID to evaluate the presence of ADHD. Cognitive evaluation was conducted with the age appropriate versions of the Wechsler Intelligence Scale^8,9^. The study protocol was approved by the Sheba Medical Center Review Board. Written informed consent was obtained from all participants after the nature of this study was explained to the subjects.

### Molecular testing for 22q11.2 microdeletion

To confirm 22q11.2 microdeletion all family members with suspicious 22q11.2DS phenotype were tested by fluorescence in situ hybridization (FISH), using the LSI TUPLE1 (*HIRA*) or N25 commercial probes (Vysis Inc., Downers Grove, IL, USA), and by multiplex ligation probe amplification (MLPA) using SALSA MLPA P250-A1 DiGeorge kit (MRC-Holland, Amsterdam, Netherlands) analyses. In addition comparative-genomic hybridization (CGH) analysis, using Illumina HumanOmni Express-12 v 1.1 BeadChip (Illumina Inc, San Diego, CA) was conducted for all 10 family members (grandmother, parents and 7 children).

### Genotyping in 22q11.2 region

*COMT* Val158Met polymorphism (rs4680) was genotyped by the C25746809-50 TaqMan kit (Applied Biosystems Incorporated, Foster City, CA) using the ABI 7000 instrument. The genotyping of the seven additional SNPs from *COMT-ARVCF* region (rs2097603, rs4633, rs4818, rs3838146, rs165599, rs2073748, rs2240717) is described in our previous study^10^ and the three short tandem repeats (STRs) markers (D22S1648, D22S941, and D22S944) were genotyped by Morrow et al.^11^ and Perez et al.^12^. The genotyping of the two *PRODH* functional SNPs rs2008720 and rs4819756 were described in our previous studies^13,14^. The additional nine *PRODH* SNPs from exon 12 (rs16983466, rs2238731, rs2904552, rs2904551, rs3970559, rs2238730, rs2904550, rs2870984, and rs2870983) were performed by PCR amplification of a 452 bp fragment (primers: F-5′gggacagaggttggaggc-3′; R- 5′ggacacatgtggctgacaag-3′) followed by sequencing with BigDye Terminators using an ABI PRISM 3100 Genetic Analyzer (Applied Biosystems, Foster City, USA).

### Whole-exome sequencing (WES) analysis

WES analysis of genomic DNA from 7 family members (parents and five of their children, which four of them are 22q11.2DS affected), was conducted by EdgeBio Company (EgdeBio, Gaithersburg, MD). Exon capture was performed with the Nimblegen SeqCap EZ Human Exome v3 (Roche NimbleGen, Madison, Wisconsin, USA). Paired end library was constructed using the Illumina TruSeq Sample Prep. Exon libraries were sequenced with the Illumina Hiseq 2000 platform (Illumina, San Diego, California, USA), providing about 100 bp read length for each sample with theoretical average coverage—100×. Variant tools^15^ “bwa_gatk28_hg19” pipeline which followed the “Best GATK (v2.8-1-g932cd3a) practice” was used for read alignment and variant calling. Briefly, sequencing data were aligned to the human reference genome build 37 using the Burrows Wheeler Alignment tool (BWA 0.7.7) with default parameters. Duplicated reads were marked with Picard tool (http://broadinstitute.github.io/picard/), and local realignment of reads around indels was performed in order to avoid misalignments due to indels. Next, base quality scores were recalibrated in order to get more accurate base qualities, which in turn improve the accuracy of the variant calls. Raw variants were called with the GATK’s UnifiedGenotyper following SNP and indels recalibration process which assigned a well-calibrated probability to each variant call. Filtering was done based on this score, and only variant calls which assigned the “PASS” filter (see GATK documentation for more details) were considered in downstream analysis. Variants were annotated against the single nucleotide polymorphism database (dbSNPs v.138), the 1000 Genome database and the RefSeq database.

### Bioinformatic databases and tools

The analysis of the WES data started from 1,215,052 variants, including exonic, 5′/3′ UTR, intergenic, intragenic and splice-sites. Filtering for only exonic variations revealed 27,988 variants out of which 13,854 were nonsynonymous.

The validation of WES results for selected candidate variants were performed using SNP genotyping using the Sequenom Mass Array platform^16^ and Sanger sequencing. Seven SNPs within the 22q11.2 were validated using TaqMan and RFLP analysis methods.

Predicted level of variant penetrance was obtained using SIFT (http://sift.jcvi.org/)^17^ and Polyphen (http://genetics.bwh.harvard.edu/pph2/)^18^. VarElect (http://varelect.genecards.org/)^19^, DisGeNET (http://www.disgenet.org/web/DisGeNET/menu)^20^, and Schizophrenia Exome Sequencing Genebook^2^ were also used to characterize variations. All genomic data for molecular variants in this study were compatible with Genome build GRCh37. NCBI gene database (https://www.ncbi.nlm.nih.gov/gene), Ensembl^21^, Exome aggregation consortium (ExAC)^22^, Vertebrate Genome Annotation database (Vega; http://vega.sanger.ac.uk/index.html)^23^, and UCSC (https://genome.ucsc.edu/)^24^ were used for genome builds and general gene and SNP (rs ID) annotations.

Database of genomic variants (DGV; http://dgv.tcag.ca/dgv/app/home)^25^ and Integrative Genomics Viewer (IGV; http://software.broadinstitute.org/software/igv/)^26^ were used for SNP analysis. Functional and pathway of specific genes resulting from WES and CGH-array were analyzed using DAVID (The Database for Annotation, Visualization and Integrated Discovery) Functional Annotation Bioinformatics Microarray Analysis (https://david.ncifcrf.gov/)^27^. KEGG database was used to investigate biological pathways^28^.

Protein–protein interaction analysis of selected genes was performed using STRING database (http://string-db.org/)^29^ and GeneMania (http://genemania.org/)^30^.

## Results

### De novo and inherited rare CNVs contribution

CGH-array analysis was performed for searching of de novo and rare CNVs and determination of the deletion borders in 10 family members (Fig. 1) using Illumina HumanOmni Express-12 v 1.1 BeadChip. Fifteen CNVs were detected in this family (Supplementary Table 2). De novo CNVs (17q21.31-gain and 11q14.1-loss) were identified only in the SZ-VC625 individual. Out of the 13 parental CNVs, rare paternal 3p26.3-loss (DGV^25^ frequency 0.06%) was transmitted to all 22q11.2DS offspring and another one, rare maternal 14q11.2-gain (DGV frequency 0.09%), to the SZ sibling. Relatively high rates of parental CNVs were found in the SZ-VC625 (5/13) and DD-VC624 (7/13) siblings in comparison to their non-psychotic sister (1/13) (Supplementary Table 2).

### 22q11.2-haplotype association with psychosis

To examine the association between 22q11.2 intact region and psychosis, we performed haplotype analysis using 73 polymorphisms from the deleted region (chr22:18,900,750-21,350,369; GRCh37): 51 variants, covered by WES and filtered by GATK “PASS” filter (see Materials and methods), and 22 additional variants by TaqMan, PCR, RFLP and Sanger sequencing. The haplotype analysis revealed transmission of a “risk” haplotype from the father to the three psychotic offspring, while the putative “protective” haplotype was transmitted to their non-psychotic daughter. These two haplotypes differed in 42 polymorphic sites out of the 73 (Supplementary Table 3). The “risk” haplotype encompassed two “damaging” variants, *PRODH*-rs2904552/T and *CLTCL1*-rs1061325/C, that are located on histone binding site, including H3K27me3 and H3K36me3 modifications (http://www.mutationtaster.org/)^31^. These two variants were not presented in the non-psychotic mother (VC622) and daughter (VC621) that carried different haplotypes (Supplementary Table 3).

### SZ gene-set and putative pathways suggested by WES and CGH-array bioinformatic analysis

**Fig. 2:**
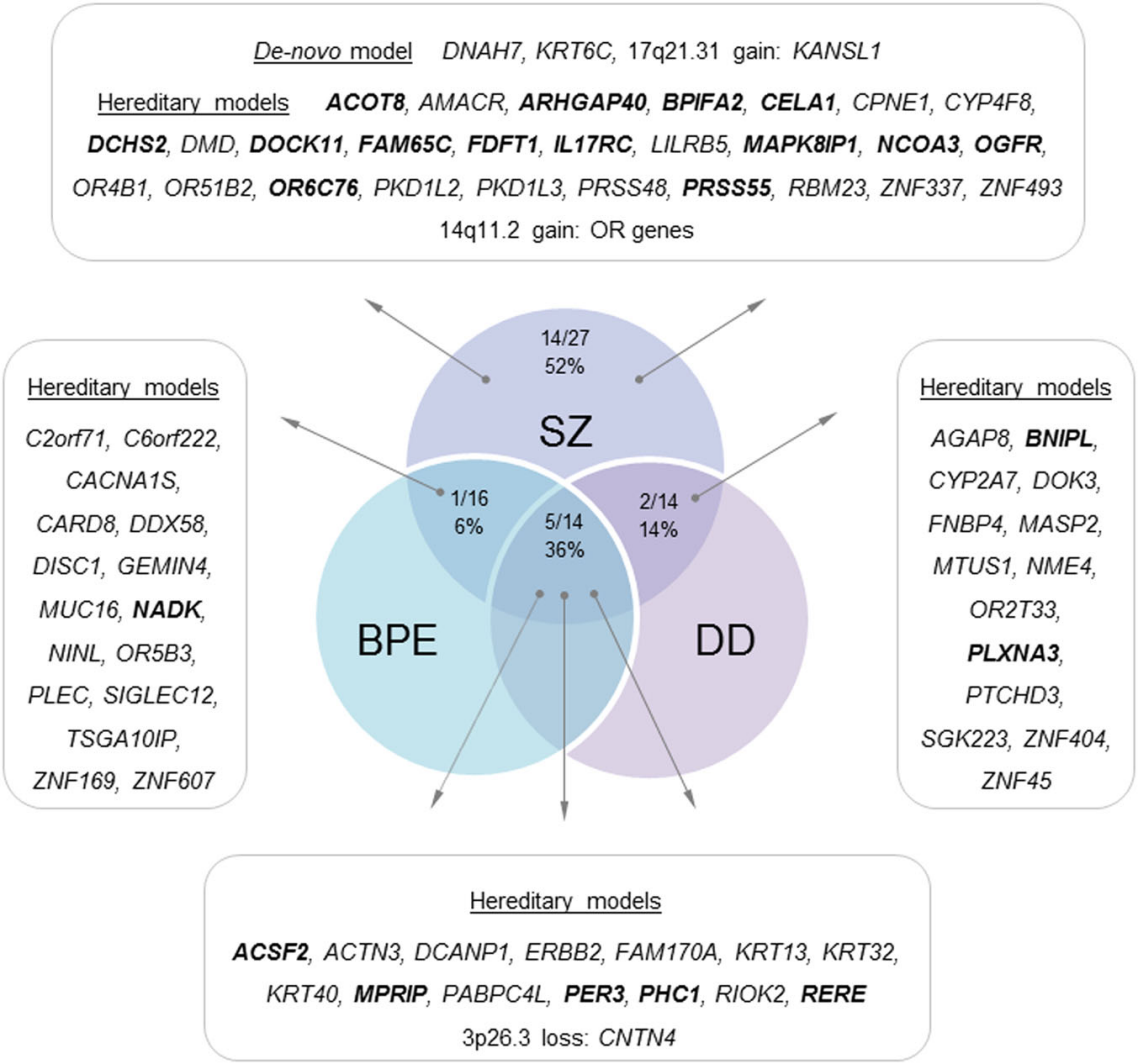
Genetic contributors to SZ-spectrum disorders in the 22q11.2DS family. Genes with damaging variants that are common to the three psychotic siblings as well as those unique to the SZ sibling are presented. The damaging variants were selected using SIFT and Polyphen2 prediction (Supplementary Table 4 and 5) and overlapping with gene-set of 11 whole-exome sequencing studies in SZ and autism spectrum disorder populations (Supplementary Table 6). Genes with rare (≤0.05) variants, excluding de novo and CNVs, are bolded and presented in rates. BPE brief psychotic episode, DD delusional disorder, OR olfactory receptor genes, *OR4K1, OR4K2, OR4K5, OR4K15, OR4M1, OR4N2*, and *OR4Q3*, covered 14q11.2-gain, SZ schizophrenia

To search for de novo, rare/damaging variants, common to the three psychotic siblings as well as those unique for SZ-VC625 bioinformatic selection analysis (PolyPhen2^18^ and SIFT^32^ tools) of 1,215,052 WES variants were conducted using recessive, de novo, and Venn tool models. The results of the bioinformatic selection are presented in Supplementary Tables 4 and 5. Following the bioinformatics selection, a further screening was conducted by examining the overlap with gene-sets established by eleven WES^2,33–37^ studies in SZ, including 22q11.2DS-related SZ^36^, and autism spectrum disorder (ASD)^38–41^ (Supplementary Table 6). The putative genetic contributors that were common to the three psychotic siblings and unique for SZ individual in the studied family, based on WES-bioinformatic selection and CGH-array are presented in a Venn diagram with emphasis on the rare (≤0.05) and de novo variants (Fig. 2).

### Candidates common to the psychosis phenotype

CGH-array identified a 3p26.3 rare deletion (DGV)^25^ transmitted from the father to the three psychotic siblings. This CNV includes *CNTN4* gene that has a role in formation of axon connections, neuronal network formation/plasticity and synaptogenesis in the developing nervous system (GeneCards: https://www.genecards.org/). In addition, 14 variants with damaging prediction (SIFT and Polyphen2) were selected as common to psychosis phenotype by WES-bioinformatic analysis (Supplementary Table 4). Five of them (36%), located in *ACSF2*, *MPRIP*, *PER3*, *PHC1*, and *RERE* genes, were rare variants (Fig. 2, Supplementary Table 4). *RERE* and *PER3* are located in the same 1p36.23 locus. *RERE* and *PHC1* are transcriptional repressors involved in chromatin organization and histone modifications during embryonic development. *PHC1* is associated with microcephaly and intellectual disability (DisGeNET). *PER3* is also a transcription factor that involved in circadian entrainment. Three keratin intermediate filament candidate genes (*KRT13*, *KRT32*, and *KRT40*, Fig. 2), clustered on 17q21, also related to circadian entrainment superpathway. Interestingly, *ACSF2* that involved in fatty acyl-CoA biosynthesis is located on the same 17q21 locus. Another cytoskeleton-related candidate, *MPRIP*, regulates disassembly of stress fibers in neuronal cells (GeneCards).

### Candidates for SZ phenotype and pathway enrichments

In addition to the candidates common for psychosis phenotype, we attempted to identify genetic factors unique to the SZ patient. Using de novo, recessive and Venn tool models additional variants with damaging SIFT/Polyphen2 predictions were selected (Supplementary Table 5). De novo CNVs, as described in the above section, were detected only in the SZ-VC625 sibling. Interestingly, 17q21.31-gain is located on 17q21 region that covered candidate genes common to psychotic siblings, namely, *ACSF2* and keratin genes. In addition, two de novo missense mutations, located in the *DNAH7* and *KRT6C* genes, were detected in the SZ sibling (Supplementary Table 5). All bioinformatically selected genetic contributors, including de novo and heritable, common to psychosis phenotypes and unique to SZ, are presented in the Fig. 2. As presented in this figure, the rate of the heritable rare variants (excluding CNV variants) that were unique for the SZ sibling was 52% (14/27), whereas the rate of the rare variants common to the three psychotic siblings was 36% (5/14).

**Fig. 3:**
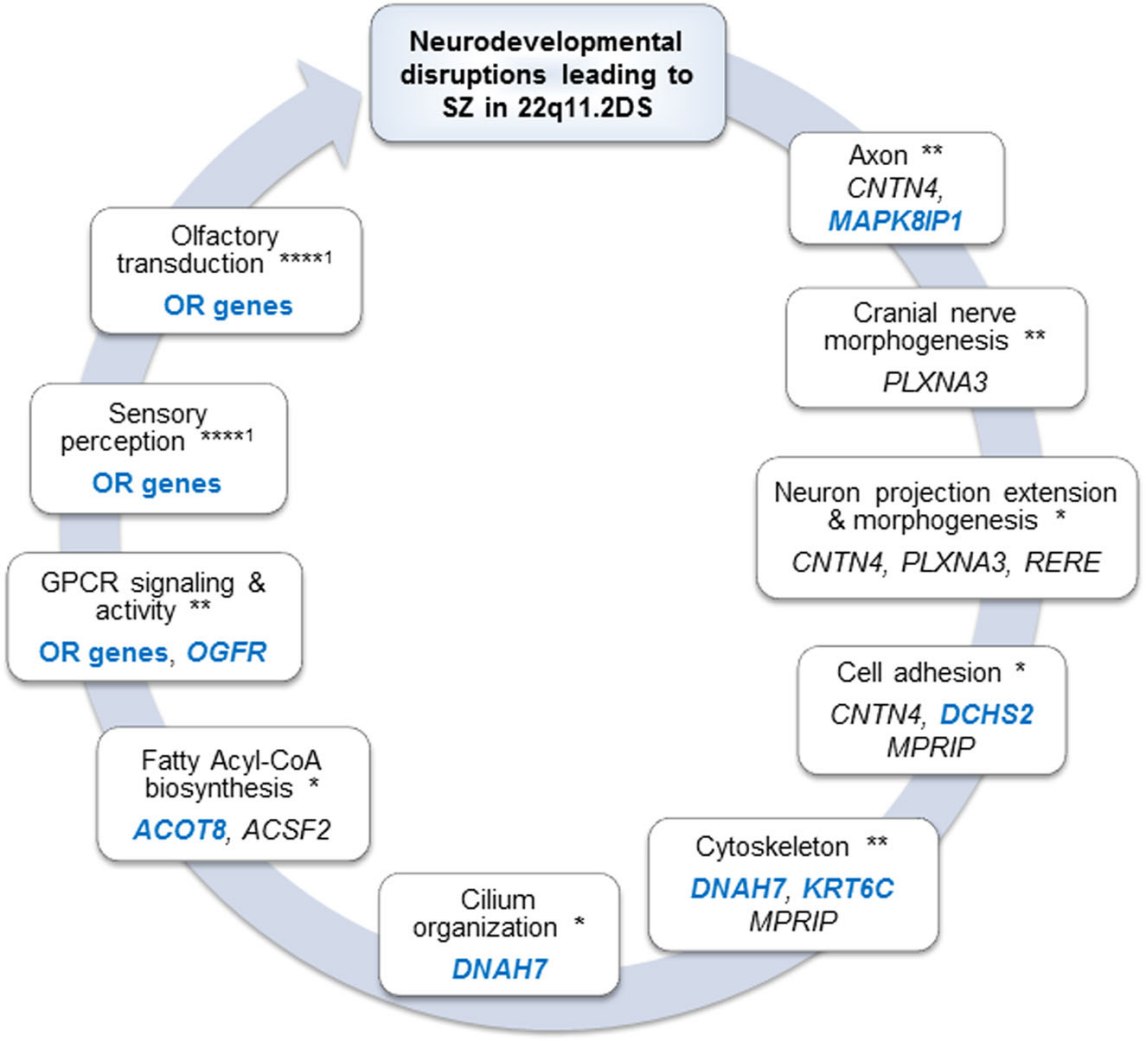
DAVID functional candidate genes enrichment for the SZ patient. Only genes with de novo and rare (≤0.05) variants are presented. Full list of bioinformatically selected candidates are presented in Supplementary table 7. Genes with variants, unique for SZ-VC625, are colored in blue. *p*-value for enrichment: *p ≤ 0.05; **p ≤ 0.01; ***p ≤ 0.005; ****p ≤ 0.00001^1^. functional enrichments remained significant following Benjamini correction. GPCR G-protein coupled receptor, OR olfactory receptor genes contained rare variants unique to the SZ sibling: *OR6C76* and seven additional, *OR4K1, OR4K2, OR4K5, OR4K15, OR4M1, OR4N2* and *OR4Q3*, covered 14q11.2-gain

The 120 candidates selected for the SZ sibling (Supplementary Table 6), including 28 genes from the 22q11.2 deleted region and 82 additional from the entire genome, were used for running the DAVID^27^ functional enrichment analysis. The statistically significant DAVID functional enrichments are presented in Fig. 3 (details in Supplementary Table 7).

Three signaling pathways, including transmembrane signaling receptor activity, detection of stimulus involved in sensory perception and olfactory transduction remained significant (*p* < 0.04) following Benjamini correction for multiple testing. The G-protein-coupled receptor (GPCR) signaling and activity also revealed significant results (*p* < 0.009) but did not survive after post hoc correction. Additional significant DAVID clustering identified neurodevelopmental pathways, including cranial nerve development/morphogenesis and neuron projection extension/morphogenesis. It is of note that the majority (~80%) of the genes enriched for sensory perception, olfactory transduction, and GPCR signaling were consisted of genes unique to the SZ patient, while the majority (75–88%) of the genes contributing to neurodevelopmental processes, including neuron projection extension/morphogenesis, were common to the psychosis phenotype (Supplementary Table 7).

The histone modification processes did not appear to be significantly enriched by DAVID analysis. However, genes involved in these process included de novo (*KANSL1*) and inheritable (*CNTN4, RERE*, *NCOA3*, and *PHC1*) rare mutations (Supplementary Table 7). Specifically, *RERE*, *PHC1* and *CNTN4* variants were common to the three psychotic siblings and *KANSL1* and *NCOA3* variants were unique for SZ patient. In addition, fatty acyl-CoA biosynthesis and charge relay system processes, which involved in histone acylation and deacethylation, revealed DAVID statistical significance (*p* = 0.02 and *p* = 0.03, respectively) (Supplementary Table 7).

## Discussion

The goal of this study was to search for “second hits” that may contribute to the development of psychosis and SZ in a unique multiplex family with high load of psychotic expression affected by the inherited 22q11.2 deletion.

### Candidates common to psychosis phenotype

Firstly, the putative contribution of the 22q11.2 intact chromosome to psychosis phenotype was examined. In the analyzed pedigree, one of the interesting finding is that the same haplotype in the 22q11.2 region is transmitted by the unaffected father to each of the three psychotic offspring. The corresponding 22q11.2-haplotype analysis revealed an association between a specific “risk” haplotype, contained two “damaging” variants in *PRODH* and *CLTCL1* genes, and psychosis. The identified rare and damaging *PRODH*-rs2904552 variant as well as *CLTCL1* de novo mutation were previously reported to be associated with SZ^42,43^. The *CLTCL1* has a role in synaptic plasticity (KEGG:hsa04721), neural crest development^44^, neuronal depolarization^45^, and histone modification^46^. Two previous case–control studies reported that variants on the 22q11.2 intact chromosome are not major contributors to SZ^36^ and psychosis^47^. The haplotype analysis of large multiplex families, in contrast to case–control studies, enables to examine contribution of the 22q11.2 gene variants, inherited together as a haplotype, to psychosis using internal control for comparison that eliminates the need for population stratification.

When a CNV is not de novo, the pedigree offers an opportunity to identify other genetic factors than the 22q11.2-loss, “second hits”, contributing to the transmission of psychosis. Bioinformatic analysis of WES and CGH-array data selected 15 common for psychosis phenotype damaging variants and 6 of them were rare mutations, including paternal 3p26.3-loss and 5 additional missense mutations located in *ACSF2, MPRIP, PHC1*, *PER3*, and *RERE* genes. It is of note that the identified candidates were previously reported to be involved in neurodevelopmental processes and to be associated with psychosis and SZ. The 3p26.3-loss, that covered *CNTN4*, was previously reported to be associated with psychosis in 22q11.2DS^48^. *CNTN4* gene is involved in synaptic plasticity^49^, prefrontal neuron-specific histone methylation^50^, response to antipsychotics^51^, and was implicated in both SZ^52^ and autism^53^. *RERE* and *PER3* are located on the 1p36.23, one of the reported 108 SZ-associated loci^3^. *RERE* and *PHC1* are transcriptional repressors involved in chromatin organization and histone modifications during embryonic development. PER3 is a transcriptional factor that together with the three keratin genes, established as candidates to psychosis in the current study, is related to circadian entrainment superpathway, recently reported as an overlapped superpathway for SZ, ASD, and bipolar disorder (BD)^54^. Interestingly, four candidates for psychosis (three keratin genes and *ACSF2*), identified in the current study, are located on 17q21 locus that was suggested to be associated with SZ in a linkage study that analyzed 175 families with at least two siblings suffering from SZ/SZ-affective disorders^55^.

### Candidates unique to SZ phenotype and functional enrichment

The next step of the study focused on search for risk gene-set and putative pathways that may contribute to the development of SZ. The detection of de novo mutations in the SZ sibling including CNVs (17q21.31-gain and 11q14.1-loss) and missense mutations (*DNAH7* and *KRT6C*) supports a de novo mutational paradigm for SZ^37,56,57^. The 17q21.31-gain may be a relevant SZ risk factor since it contains the neuronal chromatin modifier *KANSL1* gene^58^ and causes microduplication syndrome expressed in psychomotor retardation, poor social interaction and intellectual disability^59^. In addition, 17q21.31-gain was established in childhood onset SZ^60^. Notably, the 17q21 region was demonstrated in the present study to be associated as a risk region common to the three psychotic siblings and as discussed above was previously reported to be associated with SZ^55^. The 11q14.1-loss did not contain coding genes, however in 1.6 Mb upstream to this loss a rare deletion was previously reported in a patient with sporadic SZ^57^, and the same region was found to be highly associated (LOD = 3.44) with BD in a linkage study^61^.

DAVID^27^ functional enrichment analysis that was performed for bioinformatically selected SZ gene-set, identified several statistically significant biological pathways and processes. Sensory perception and neuron projection extension/morphogenesis established in the current study are consistent with previously reported findings in 22q11.2DS-related SZ^36^. The GPCR signaling and olfactory transduction pathways, revealed in the current study, were previously reported to be involved in idiopathic^37,62^ and early-onset^63^ SZ, respectively.

Histone modification process, implicated in SZ in a large (60,000 participants) GWAS study^64^ did not appear to be significantly enriched by DAVID analysis, however, the candidate genes involved in this process were affected by rare de novo and inherited variants. These variants include CNVs covered two histone modification genes, *KANSL1*^58,65^ (17q21.31-gain) and *CNTN4*^50^ (3p26.3-loss), and missense mutations in *RERE*, *NCOA3* and *PHC1* genes. Moreover, “fatty acyl-CoA biosynthesis” and “charge relay system” processes, which were previously reported to be involved in histone acylation and deacethylation^66,67^, exhibited DAVID significant results.

The major weakness of this study is the small sample size of the pedigree, which limits the potential of reaching statistical significance. However, the studied pedigree is unique multiplex 22q11.2DS family with high load of psychosis expression comprised of eight offspring which half of them inherited the maternal deletion. In addition, the fact that one of the three siblings was diagnosed with SZ enabled us to attempt to identify both, variants common to the psychosis phenotype as well as variants that are unique to the SZ individual.

Thus, it appears that the strength of our pedigree design is that all cases share the same genetic causes identical by descent (IBD) that enabled the identification of molecular events relevant to the psychosis phenotype. Furthermore, the value of the pedigree is the exclusion of events not shared by all the affected offspring.

We suggest that specific molecular events, CNVs and missense mutations, outside the 22q11.2, account for disruptions in neurodevelopmental processes, including neuron projection extension and morphogenesis, cytoskeleton signaling and histone modification, that may contribute to the psychosis phenotype in 22q11.2DS. Apparently, the combination of a larger load of damaging molecular events with greater harmful effects on neurodevelopmental processes, together with additional effects on sensory perception, olfactory transduction and GPCR signaling may explain development of SZ in 22q11.2DS. A future study including large 22q11.2DS families with SZ and non-SZ psychosis, as well as a comparison sample of large families with non-22q11.2DS SZ may help in establishing biological pathways relevant to the pathophysiology of SZ and may lead to the development of novel treatments.

## Supplementary information

Supplementary Tables
