# Supplementary material for: Risk gene-set and pathways in 22q11.2 deletion-related schizophrenia: a genealogical molecular approach
Source: Transl Psychiatry. 2019 Jan 17;9:15. doi: 10.1038/s41398-018-0354-9 (PMC6358611; doi:10.1038/s41398-018-0354-9)
Supplement: Supplementary file 1 — Supplementary Tables [file 41398_2018_354_MOESM1_ESM.docx]

Supplementary Table 1

**Clinical characteristics of the 5 affected family members with 22q11.2DS**

| Subject no. | Gender | Age* (yrs) | Education (yrs) | Medical  characteristics | Cognitive Level (FSIQ) | Psychiatric Disorders | Marital Status | Employment |
| --- | --- | --- | --- | --- | --- | --- | --- | --- |
| VC621 | F | 35 | 12 | Rt. Conductive hearing loss; Eosinophilia; Hypotonia; Nearsightedness; Nasopharyngeal disproportion; Occult submucous cleft palate | 66 | GAD | Married | Yes |
| VC622 | F | 68 | 10 | Anemia; Hypotonia; Nearsightedness; Rampant caries-no teeth | 60 | Specific phobia; ADHD-Combined type | Married | No |
| VC624 | M | 43 | 8 | Vitiligo; umbilical and epigastric hernias; Conductive hearing impairment; Hypotonia; Left amblyopia; Caries; Nasal septum deviation | 59 | DD | Married | Yes |
| VC625 | M | 42 | 12 | Aberrant subclavian artery from ascending aorta; Hypotonia; Scoliosis | 56 | SZ | Single | No |
| VC629 | F | 25 | 12 | Aberrant right subclavian artery; Foramen ovale aneurysm; Factor VII deficiency; Umbilical and epigastric hernias; Hearing loss; Pes valgus; Hypotonia; Nearsightedness; Astigmatism; Caries; Scoliosis | 58 | BPE; GAD; ADHD-Inattentive presentation,  Specific phobia and social anxiety disorder | Single | No |

* Age at psychiatric diagnosis.

ADHD - attention deficit/ hyperactivity disorder; BPE - brief psychotic episode; DD - delusional disorder; GAD - generalized anxiety disorder; SZ – schizophrenia

Supplementary Table 2 **CNVs detected by CGH-array in 22q11.2DS family**

| Genomic location  (GRCh37) | VC6222 Grand  mother | VC622 Mother  Non-Psych | VC623 Father | VC624  Psych  DD | VC625  Psych  SZ | VC629  Psych  BPE | VC621  Non-Psych | VC626 | VC627 | VC628 |
| --- | --- | --- | --- | --- | --- | --- | --- | --- | --- | --- |
| 1p36.33 gain  930,646-1,292,073 |  |  | 33 genes |  |  |  |  |  |  |  |
| 1q21.2 gain  148,937,908-149,201,987 |  |  | *NBPF25P, LOC*  *645166* | *NBPF25P, LOC*  *645166* | *NBPF25P, LOC*  *645166* |  |  |  |  |  |
| 3p26.3 loss  2,184,249-2,225,547 | *CNTN4-AS2,*  *CNTN4* |  | *CNTN4-AS2,*  *CNTN4* | *CNTN4-AS2,*  *CNTN4* | *CNTN4-AS2,*  *CNTN4* | *CNTN4-AS2,*  *CNTN4* | *CNTN4-AS2,*  *CNTN4* |  | *CNTN4-AS2,*  *CNTN4* |  |
| 7p12.1 loss  53,461,805-53,596,752 |  | NCG |  | NCG | NCG |  |  |  |  |  |
| 8q24.23 loss  137,681,144-137,866,864 | NCG |  | NCG |  | NCG |  |  |  |  |  |
| **11q14.1 loss** 81,504,727-81,521,487 |  |  |  |  | **NCG** |  |  |  |  |  |
| 12p13.31 gain  7,997,114-8,118,867 |  |  | 5 genes |  |  |  |  |  |  |  |
| 13q13.3 loss  38,069,399-38,119,814 |  |  | 1 gene |  |  |  |  |  |  |  |
| 14q11.2 gain  20,213,937-20,445,494 | 7 olfactory genes^1^ | 7 olfactory genes^1^ |  |  | 7 olfactory genes^1^ |  |  |  |  |  |
| 15q14 loss  34,715,886-34,835,945 |  |  | 4 genes |  |  |  |  |  |  |  |
| 16p13.3 gain  1,806,040-1,846,062 | *MAPK8IP3 NME3 MRPS34 EME2 SPSB3 NUBP2 IGFALS* | *MAPK8IP3 NME3 MRPS34 EME2 SPSB3 NUBP2 IGFALS* | *MAPK8IP3 NME3 MRPS34 EME2 SPSB3 NUBP2 IGFALS* | *MAPK8IP3 NME3 MRPS34 EME2 SPSB3 NUBP2 IGFALS* |  |  |  |  |  |  |
| 17p12 loss 15,042,166-15,058,613 |  |  | NCG | NCG |  |  |  |  |  |  |
| **17q21.31 gain** 44,166,866-44,368,212 |  |  |  |  | ***KANSL1 KANSL1-AS1*** |  |  |  |  |  |
| 19p12 loss  20,563,347-20,717,095 | *ZNF826P, MIR1270-1* |  | *ZNF826P, MIR1270-1* | *ZNF826P, MIR1270-1* |  |  |  |  |  |  |
| 22q11.2 deletion  18,844,632-21,703,145 |  | 48 genes^2^ |  | 48 genes^2^ | 48 genes^2^ | 48 genes^2^ | 48 genes^2^ |  |  |  |
| 22q12.3 loss 34,556,204-34,583,646 |  | LL22NC03-86D4.1 |  | LL22NC03-86D4.1 |  |  |  |  |  | LL22NC03-86D4.1 |
| Total CNVs (n=15) | 5 | 4 | 10 | 7 | 7 | 1 | 1 | 0 | 1 | 1 |

All individuals with 22q11.2DS are highlighted in color. *De novo* CNVs are bolded.

^1^ - *OR4Q3, OR4M1, OR4N2, OR4K2, OR4K5, OR4K1, OR4K15*; ^2^ - Chr22:18,844,632-21,703,145 deleted region contains 115 genes, including 48 protein coding genes (limited by *DGCR6* and *GGT2*), 7 miRNA, 10 non-protein coding genes/RNA, 35 pseudogenes and 15 uncharacterized sequences (UCSC: <https://genome.ucsc.edu/index.html>).

Abbreviations: BPE-brief psychotic episode; DD-delusional disorder; NCG -no coding genes; Non-Psych-no psychosis; Psych-psychosis; SZ-schizophrenia

Supplementary Table 3

**Association of a specific 22q11.2 haplotype with psychotic phenotype in the 22q11.2DS family**

| Gene Symbol N=20 | Chr Position GRCh37 | Rs ID  N=42 | SIFT | Polyphen2  prediction | GMAF | Variant type | VC623 Father | | VC622 Mother | VC621 Non-Psych | VC624 Psych | VC625 Psych | VC629 Psych |
| --- | --- | --- | --- | --- | --- | --- | --- | --- | --- | --- | --- | --- | --- |
| *PRODH* | 18905964 | rs2904552 | 0.04 | D/D | 0.04 | nonsyn | C | T | C | C | T | T | T |
| *PRODH* | 18908875 | rs1808320 |  |  | 0.70 | syn | A | G | G | A | G | G | G |
| *PRODH* | 18912678 | rs4819756 | 0.53 | B/B | 0.73 | nonsyn | A | G | G | A | G | G | G |
| *PRODH* | 18923745 | rs2008720 |  | B/B | 0.44 | nonsyn | G | T | T | G | T | T | T |
| *DGCR14* | 19132061 | rs2298274 |  |  | 0.08 | syn | T | C | C | T | C | C | C |
| *SLC25A1* | 19165478 | rs2070255 |  |  | 0.08 | syn | G | A | A | G | A | A | A |
| *CLTCL1* | 19168288 | rs5748024 | 0.018 | B/B | 0.01 | nonsyn | T | C | C | T | C | C | C |
| *CLTCL1* | 19170956 | rs2073738 | 1 | B/B | 0.03 | nonsyn | T | C | C | T | C | C | C |
| *CLTCL1* | 19184095 | rs1061325 | 0.14 | D/D | 0.4 | nonsyn | T | C | T | T | C | C | C |
| *CLTCL1* | 19230365 | rs5746697 | 0.47 | B/B | 0.02 | nonsyn | C | T | T | C | T | T | T |
| *CLTCL1* | 19241488 | rs190950286 |  |  | 0.004 | syn | T | C | C | T | C | C | C |
| *CLTCL1* | 19263214 | rs3747059 | 0.17 | D/D | 0.02 | nonsyn | A | G | G | A | G | G | G |
| *STR* | 19407085-19407256 | D22S1648 |  |  |  | STR | 4 | 3 | 3 | 4 | 3 | 3 | 3 |
| *STR* | 19409184-  19409409 | D22S941 |  |  |  | STR | 1 | 9 | 9 | 1 | 9 | 9 | 9 |
| *MRPL40* | 19423250 | rs7575 | 1 | B/B | 0.21 | nonsyn | A | G | G | A | G | G | G |
| *CDC45* | 19468514 | rs13447189 |  |  | 0.02 | syn | C | T | T | C | T | T | T |
| *CLDN5* | 19512918 | rs5748258 |  |  | 0.05 | int | T | C | C | T | C | C | C |
| *STR* | 19608513-19608706 | D22S944 |  |  |  | STR | 6 | 4 | 3 | 6 | 4 | 4 | 4 |
| *TBX1* | 19751829 | rs2301558 |  |  | 0.29 | syn | C | T | C | C | T | T | T |
| *GNB1L* | 19808769 | rs5748449 | 0.52 | B/B | 0.13 | nonsyn | T | C | T | T | C | C | C |
| *GNB1L* | 19839438 | rs17745302 | 0.11 | B/B | 0.03 | nonsyn | C | T | C | C | T | T | T |
| *TXNRD2* | 19867771 | rs1139795 |  |  | 0.25 | syn | C | T | T | C | T | T | T |
| *TXNRD2* | 19868218 | rs1139793 | 0.15 | B/B | 0.30 | nonsyn | A | G | G | A | G | G | G |
| *TXNRD2* | 19882984 | rs5992495 | 0.34 | B/B | 0.33 | nonsyn | G | T | G | G | T | T | T |
| *TXNRD2* | 19906511 | rs11541479 |  |  | 0.16 | syn | A | G | G | A | G | G | G |
| *TXNRD2* | 19907118 | rs5748470 |  |  | 0.60 | syn | A | G | A | A | G | G | G |
| *COMT* | 19950235 | rs4633 |  |  | 0.39 | syn | T | C | T | T | C | C | C |
| *COMT* | 19951207 | rs4818 |  |  | 0.30 | syn | C | G | C | C | G | G | G |
| *COMT* | 19951271 | rs4680 | 0.06 | B/B | 0.37 | nonsyn | A | G | A | A | G | G | G |
| *COMT* | 19956262 | rs362204 |  |  | 0.40 | UTR-3 | C | delC | C | C | delC | delC | delC |
| *COMT* | 19956781 | rs165599 |  |  | 0.50 | UTR-3 | A | G | A | A | G | G | G |
| *ARVCF* | 19969075 | rs2073747 |  |  | 0.72 | syn | G | A | G | G | A | A | A |
| *ZDHHC8* | 20131115 | rs9605069 |  |  | 0.08 | syn | C | T | C | C | T | T | T |
| *SCARF2* | 20784050 | rs2241230 | 0.55 | B/B | 0.22 | nonsyn | T | A | T | T | A | A | A |
| *KLHL22* | 20800835 | rs8748 |  |  | 0.20 | syn | A | G | A | A | G | G | G |
| *MED15* | 20940085 | rs2285699 |  |  | 0.14 | syn | G | A | G | G | A | A | A |
| *PI4KA* | 21134023 | rs35646566 |  |  | 0.01 | syn | A | G | G | A | G | G | G |
| *PI4KA* | 21141300 | rs4675 |  |  | 0.55 | syn | T | C | C | T | C | C | C |
| *PI4KA* | 21167787 | rs165854 |  |  | 0.52 | syn | G | A | A | G | A | A | A |
| *SNAP29* | 21213416 | rs1061064 |  |  | 0.39 | syn | G | A | A | G | A | A | A |
| *CRKL* | 21272616 | rs75659674 |  |  | 0.01 | int | T | G | G | T | G | G | G |
| *LZTR1* | 21350369 | rs117346988 |  |  | 0.0014 | syn | C | T | C | C | T | T | T |

The “risk” and “protective” haplotypes are colored in purple and blue, respectively. Underlined variants were validated. The other variants have good call reads (See Methods). The alternative allele is underlined. Blank cells represent unavailability of data. Abbreviations: GMAF-global allele frequency; Polyphen2 HDIV/HVAR prediction: B-benign, D-probably damaging; Variant type: Nonsyn - non synonymous; Syn-synonymous; STR-short tandem repeats; int - intronic; UTR-3 – untranslated region. Non psych- no psychosis; Psych – psychosis

Supplementary Table 4

**Candidate variants for psychosis phenotypes in the 22q11.2DS family: Venn tool model**

| Variant carriers | Gene symbol | Cytoband | Chr position GRCh37 | Rs ID | Variant  Ref/Alt | Exonic Function | SIFT score | Polyphenprediction | GMAF MAF/AF | VarElect algorithm |
| --- | --- | --- | --- | --- | --- | --- | --- | --- | --- | --- |
| **All psychotic siblings** | ***ACSF2*** | 17q21.33 | 48548419 | rs142467151 | G/A | nonsyn | 0 | D/P | 0.0005 |  |
|  | *ACTN3* | 11q13.2 | 66328741 | rs2229456 | A/C | nonsyn | 0 |  | 0.16 |  |
|  | *DCANP1* | 5q31.1 | 134782450 | rs12520799 | T/A | stopgain | 0 |  | 0.35 |  |
|  | *ERBB2* | 17q12 | 37884037 | rs1058808 | C/G | nonsyn | 0.03 | P/P | 0.49 | SZ,BD,ASD |
|  | *FAM170A* | 5q23.1 | 118969960 | rs328694 | C/T | nonsyn |  | D/D | 0.43 |  |
|  | *KRT13* | 17q21.2 | 39659913 | rs9891361 | G/A | nonsyn | 0.02 | D/P | 0.73 |  |
|  | *KRT32* | 17q21.2 | 39622068 | rs2071561 | G/T | nonsyn | 0 | D/D | 0.47 |  |
|  | *KRT40* | 17q21.2 | 39137154 | rs721958 | C/G | nonsyn | 0.05 | P/P | 0.34 |  |
|  | *KRT40* | 17q21.2 | 39139370 | rs9908304 | G/A | nonsyn | 0 | D/D | 0.34 |  |
|  | ***MPRIP*** | 17p11.2 | 17039562 | rs3833098 | CAG/- | nonfs del |  |  | NA | BD |
|  | *PABPC4L* | 4q28.3 | 135121066 | rs11099273 | T/G | nonsyn | 0.01 |  | 0.62 |  |
|  | ***PER3*** | 1p36.23 | 7889973 | NA | AGA^#^/- | nonfs del |  |  | NA | SZ,BD |
|  | ***PHC1*** | 12p13.31 | 9090528 | rs62625019 | G/A | nonsyn | 0.01 | D/P | 0.05 |  |
|  | ***RERE*** | 1p36.23 | 8420382 | rs779071255 | G/T | nonsyn | 0.04 | P/P | 0.00001 | SZ,BD,ASD |
|  | *RIOK2* | 5q15 | 96513471 | rs2544773 | G/C | nonsyn | 0 | D/D | 0.36 |  |
|  | *SLC22A25* | 11q12.3 | 62951221 | rs11231397 | C/G | nonsyn | 0.03 | D/D | 0.40 |  |
| **Siblings with SZ and DD** | *AGAP8* | 10q11.23 | 51225724 | rs200027998 | G/C | nonsyn | 0.01 | D/D | 0.37 |  |
|  | ***BNIPL*** | 1q21.3 | 151011325 | rs61751619 | C/T | nonsyn | 0 | D/D | 0.04 | SZ |
|  | *CCDC168* | 13q33.1 | 103410914 | rs12855785 | A/T | nonsyn | 0 |  | 0.38 |  |
|  | *CCDC168* | 13q33.1 | 103410782 | rs1375719 | T/C | nonsyn | 0 |  | 0.38 |  |
|  | *CYP2A7* | 19q13.2 | 41383799 | rs3869579 | G/A | nonsyn | 0 | D/D | 0.51 |  |
|  | *DOK3* | 5q35.3 | 176930172 | rs138153794 | GAG/- | nonfs del |  |  | 0.53 |  |
|  | *FNBP4* | 11p11.2 | 47788664 | rs67450550 | GGTGGT/- | nonfs del |  |  | 0.32 | SZ |
|  | *MASP2* | 1p36.22 | 11090916 | rs12711521 | C/A | nonsyn | 0.04 | P/P | 0.63 | SZ |
|  | *MTUS1* | 8p22 | 17612875 | rs3739407 | A/G | nonsyn | 0.01 | P/P | 0.69 |  |
|  | *NME4* | 16p13.3 | 450141 | rs35963490 | .-/AG | fs ins |  |  | 0.33 |  |
|  | *OR2T33* | 1q44 | 248436611 | rs10888338 | G/A | nonsyn | 0 | P/P | 0.63 |  |
|  | ***PLXNA3*** | Xq28 | 153693147 | rs149034613 | G/A | nonsyn | 0.04 | D/D | 0.00007 | ASD |
|  | *PTCHD3* | 10p12.1 | 27702725 | rs6482626 | A/G | nonsyn | 0 | D/D | 0.20 |  |
|  | *SGK223* | 8p23.1 | 8234869 | rs150979349 | .-/GCCGCT | nonfs ins |  |  | 0.60 |  |
|  | *SLC28A1* | 15q25.3 | 85438312 | rs151038463 | .-/TTG | nonfs ins |  |  | 0.26 |  |
|  | *TTC24* | 1q23.1 | 156551848 | rs6682716 | A/G | nonsyn | 0.03 |  | 0.42 |  |
|  | *ZNF404* | 19q13.31 | 44377669 | rs12977303 | G/A | nonsyn | 0 | D/D | 0.43 |  |
|  | *ZNF45* | 19q13.31 | 44418680 | rs388685 | G/C | nonsyn | 0.01 | D/D | 0.47 |  |
| **Siblings with SZ and BPE** | *C2orf71* | 2p23.2 | 29287927 | rs139768554 | .-/GCT | nonfs ins |  |  | 0.37 |  |
|  | *C6orf222* | 6p21.31 | 36287312 | rs743852 | G/C | nonsyn | 0.02 | D/D | 0.39 |  |
|  | *CACNA1S* | 1q32.1 | 201052310 | rs12742169 | A/T | nonsyn | 0.13 | P/P | 0.17 | SZ |
|  | *CAPN8* | 1q41 | 223813586 | rs35539373 | G/T | nonsyn | 0.06 | D/D | 0.39 | SZ |
|  | *CARD8* | 19q13.33 | 48737706 | rs2043211 | A/T | nonsyn | 0.05 | P/P | 0.33 |  |
|  | *CASC8* | 8q24.21 | 128428638 | rs6998061 | G/A | nonsyn | 0.05 | P/P | 0.41 |  |
|  | *DDX58* | 9p21.1 | 32526146 | rs10813831 | G/A | nonsyn | 0 | D/D | 0.19 |  |
|  | *DISC1* | 1q42.2 | 232144598 | rs821616 | A/T | nonsyn | 0.06 | D/D | 0.25 | SZ,BD,ASD |
|  | *DISC1* | 1q42.2 | 232144887 | rs821617 | A/G | nonsyn | 0 | D/D | 0.25 | SZ,BD,ASD |
|  | *FOLH1B* | 11q14.3 | 89413781 | rs10830339 | C/G | nonsyn | 0.02 |  | 0.23 |  |
|  | *GEMIN4* | 17p13.3 | 648186 | rs7813 | G/A | nonsyn | 0.01 | D/P | 0.69 |  |
|  | *GEMIN4* | 17p13.3 | 649547 | rs910925 | G/C | nonsyn | 0.03 | D/D | 0.69 |  |
|  | *KIAA1751* | 1p36.33 | 1887019 | rs28548017 | A/G | stoploss | 0 |  | 0.81 |  |
|  | *MUC16* | 19p13.2 | 9075346 | rs2591592 | T/A | nonsyn | 0 | D/P | 0.43 |  |
|  | ***NADK*** | 1p36.33 | 1684348 | rs150880809 | .-/CCT | nonfs ins |  |  | 0.037 |  |
|  | *NINL* | 20p11.21 | 25457050 | rs68078266 | CTCCCA/- | nonfs del |  |  | 0.55 |  |
|  | *OR5B3* | 11q12.1 | 58170342 | rs11229411 | C/T | nonsyn | 0 | D/P | 0.3 |  |
|  | *OR5B3* | 11q12.1 | 58170374 | rs12280114 | T/C | nonsyn | 0 | P/P | 0.3 |  |
|  | *PLEC* | 8q24.3 | 145001031 | rs55895668 | T/C | nonsyn | 0 |  | 0.45 | SZ |
|  | *SIGLEC12* | 19q13.41 | 52004792 | rs66949844 | .-/C | fs ins |  |  | 0.41 |  |
|  | *TBX10* | 11q13.2 | 67402362 | rs3758938 | T/G | nonsyn | 0 | D/D | 0.24 |  |
|  | *TRIM49* | 11q14.3 | 89531540 | rs12417980 | C/T | nonsyn |  | P/P | 0.29 |  |
|  | *TSGA10IP* | 11q13.1 | 65727301 | rs491973 | A/G | nonsyn | 0 |  | 0.46 |  |
|  | *ZNF169* | 9q22.32 | 97055310 | rs1536690 | C/T | nonsyn | 0 | D/P | 0.16 |  |
|  | *ZNF607* | 19q13.12 | 38189440 | rs958305 | T/C | nonsyn | 0.06 | D/P | 0.60 |  |

The selection of variants, excluding 22q11.2-loss, using Venn tool was performed for combinations of the three psychotic phenotypes. The selection of homozygous alternative variants common to each psychotic phenotype group was performed, excluding homozygosity for identical variants carried by the non-psychotic sister. The subsequent selection criteria were: exonic non-synonymous variants with SIFT≤0.05 and PolyPhen2 prediction of damaging (D) or possible damaging (P) variant. Ins/Del variants without SIFT and PolyPhen2 scoring were also selected. Rare variants (≤0.05) are bolded. Underlined variants were validated. Unavailability Rs and GMAF/MAF data (NA) indicated singleton variant. Blank cells represent unavailability of data.

^#^ AGAATCCATCCCATCCTACTGCCAGCGCTCTGTCCACAGGATCGCCTCCCATGA. Abbreviations: ASD-autism spectrum disorder; GMAF/AF- global/allele frequency from NCBI source; BD-bipolar disorder; BPE- brief psychotic episode; DD-delusional disorder; fs del/ins- frame shift deletion/insertion; nonfs del/ins- non-frame shift deletion/insertion; nonsyn-non synonymous; Ref/Alt –reference/alternative allele; syn-synonymous; SZ-schizophrenia; VarElect (<http://ve.genecards.org>) is algorithm that infers links between genes and phenotypes (only direct associations are presented).

Supplementary Table 5

**Unique candidate variants for the SZ sibling: *de novo*, recessive and Venn tool models**

| Gene symbol | Cytoband | Chr position GRCh37 | Rs ID | Variant Ref/Alt | Exonic Function | SIFT score | Polyphen prediction | GMAF /MAF | VarElect algorithm |
| --- | --- | --- | --- | --- | --- | --- | --- | --- | --- |
| ***DNAH7*** *de novo* | 2q32.3 | 196801440 | NA | A/G | nonsyn | 0.26 | D/D | NA |  |
| ***KRT6C*** *de novo* | 12q13.13 | 52866060 | rs11608915 | C/T | nonsyn | 0.03 | D/P | 0.38 |  |
| ***ACOT8*** | 20q13.12 | 44485953 | rs201025211 | .-/ATCT | fs ins |  |  | 0.03 | SZ |
| *AMACR* | 5p13.2 | 34004707 | rs10941112 | C/T | nonsyn | 0.05 | P/P | 0.35 | SZ, BD |
| *AMACR* | 5p13.2 | 34008100 | rs3195676 | C/T | nonsyn | 0.03 | P/P | 0.38 | SZ, BD |
| ***ARHGAP40*** | 20q11.23 | 37272512 | NA | G/A | nonsyn | 0.05 |  | NA | SZ |
| ***BPIFA2*** | 20q11.21 | 31756954 | rs17124277 | G/A | nonsyn | 0 | D/D | 0.03 |  |
| ***CELA1*** | 12q13.13 | 51723599 | rs76813052 | .-/G | fs ins |  |  | 0.00002 |  |
| *CPNE1* | 20q11.22 | 34218673 | rs12481228 | G/C | nonsyn | 0.02 | P/P | 0.09 | SZ |
| *CYP4F8* | 19p13.12 | 15730502 | rs61746468 | C/T | nonsyn | 0.01 | D/D | 0.10 |  |
| *DCHS2* | 4q31.3 | 155295076 | rs11721758 | A/G | nonsyn | 0.04 | B/B | 0.35 |  |
| *DMD* | Xp21.1 | 32380996 | rs1801187 | C/T | nonsyn | 0.29 | D/D | 0.45 | SZ,BD,ASD |
| ***DOCK11*** | Xq24 | 117676905 | rs149761078 | G/A | nonsyn | 0.01 | D/D | 0.00006 |  |
| *ERO1LB* | 1q42.3 | 236413230 | rs2477599 | T/A | nonsyn | 0.05 | P/P | 0.33 |  |
| ***FAM65C*** | 20q13.13 | 49225233 | rs146608853 | G/A | nonsyn | 0 | D/D | 0.0018 |  |
| ***IL17RC*** | 3p25.3 | 9965582 | rs75692599 | G/A | nonsyn | 0.03 | D/P | 0.01 |  |
| *LILRB5* | 19q13.42 | 54759361 | rs12975366 | T/C | nonsyn | 0.02 | D/D | 0.27 |  |
| ***MAPK8IP1*** | 11p11.2 | 45924283 | rs34420676 | C/T | nonsyn | 0.04 | B/B* | 0.004 | BD,ASD |
| ***NCOA3*** | 20q13.12 | 46256424 | rs6094752 | C/T | nonsyn | 0.15 | D/P | 0.03 | SZ,BD,ASD |
| ***OGFR*** | 20q13.33 | 61443716 | rs41309371 | G/A | nonsyn | 0.08 | D/D | 0.01 |  |
| *OR4B1* | 11p11.2 | 48239071 | rs12292056 | C/A | nonsyn | 0 | D/D | 0.09 |  |
| *OR51B2* | 11p15.4 | 5344681 | rs11036815 | G/A | nonsyn | 0.01 | D/D | 0.26 |  |
| ***OR6C76*** | 12q13.2 | 55820959 | rs57387180 | A/- | fs del |  |  | 0.009 | SZ, ASD |
| *PKD1L2* | 16q23.2 | 81249954 | rs7191351 | T/A | nonsyn | 0 | P/P | 0.47 |  |
| *PKD1L3* | 16q22.2 | 72003952 | rs35259348 | G/C | nonsyn | 0.03 |  | 0.23 |  |
| *PRSS48* | 4q31.3 | 152201019 | rs148861921 | .-/CAGGT | fs ins |  |  | 0.21 |  |
| ***PRSS55*** | 8p23.1 | 10411513 | rs142551217 | G/- | fs del |  |  | 0.02 |  |
| *SPINT4* | 20q13.12 | 44352620 | rs6017667 | G/A | nonsyn | 0 | D/D | 0.47 |  |
| *ZNF337* | 20p11.1 | 25666737 | rs926487 | C/T | nonsyn | 0 | D/D | 0.17 |  |
| *ZNF493* | 19p12 | 21606719 | rs10414834 | C/G | nonsyn | 0.02 | P/P | 0.10 |  |
| ***DCHS2*** | 4q31.3 | 155244402 | rs140019361 | TTTG/- | fs del |  |  | 0.025 |  |
| ***FDFT1*** | 8p23.1 | 11666219 | rs71711801 | TCCCAC/- | nonfs del |  |  | NA | ASD |
| *RBM23* | 14q11.2 | 23371256 | rs61680322 | .-/GGC | nonfs ins |  |  | 0.45 |  |

The candidate variants, excluding 22q11.2-loss, selection by a recessive model was based on the criteria that the parents were heterozygous whereas son with SZ was homozygous for alternative allele and his siblings, including non-psychotic sister (VC621), were not. The subsequent selection criteria were: exonic non-synonymous variants with SIFT≤0.05 and PolyPhen2 prediction of the damaging (D) or possible damaging (P) variant. Ins/Del variants without SIFT and PolyPhen2 scoring were also selected. *De novo* and rare (≤0.05) variants are bolded. Underlined variants were validated. Unavailability Rs and GMAF/MAF data (NA) indicated singleton variant. Blank cells represent unavailability of data. Abbreviations: ASD- autism spectrum disorder; BD- bipolar disorder; BPE- brief psychotic episode; DD-delusional disorder; fs del/ins- frame shift deletion/insertion; nonfs del/ins- non-frame shift deletion/insertion; nonsyn-non synonymous; Ref/Alt –reference/alternative allele; SZ-schizophrenia; VarElect (<http://ve.genecards.org>) is algorithm that infers links between genes and phenotypes (only direct associations are presented).

Supplementary Table 6

**Overlapping candidate genes identified for SZ sibling using SZ/ASD gene-sets from 11 WES studies**

| SZ risk genes in 22q11.2DS Current study | SZ risk genes in 22q11.2DS Case-control study Merico et al. 2015 | SZ *de novo* mutations Familial studies Xu et al. 2011 Gulsuner et al. 2013 Guipponi et al. 2014 Fromer et al. 2014 McCarthy et al. 2014 | SZ risk genes Case-control study Purcell et al. 2014 | ASD *de novo* mutations Familial studies Iossifov et al. 2012 ORoak et al. 2012 Neale et al. 2012 Sanders et al. 2012 | SZ associated genes NCBI data: [www.ncbi.nlm.nih.gov/gene](http://www.ncbi.nlm.nih.gov/gene) |
| --- | --- | --- | --- | --- | --- |
| *ACOT8* |  | *ACOT6* |  |  |  |
| *ACSF2* | *ACSM5* | *GNB2* |  |  | *GNB2* |
| *ACTN3* | *ACTN4* |  | *ACTN2,4* | *ACTN4* |  |
| *AGAP8* |  |  | *HSPB1* | *AGAP2* |  |
| *AMACR* | *PEX5* |  |  | *PSMG4* | *AMACR* |
| *ARHGAP40* |  | *ARHGAP11A* | *RHOBTB2* |  |  |
| *BNIPL* |  |  | *FKBP8* |  |  |
| *BPIFA2* |  | *ARHGAP12, HLA-C* | *ARHGAP17/20, HLA-C* | *ARHGAP30* | *HLA-C* |
| *C2orf71* |  |  | *ARPC3* |  |  |
| *C6orf222* |  | *C6orf222* | *C6orf222* |  |  |
| *CACNA1S* |  | *CACNA1S* |  | *CACNA1S* |  |
| *CARD8* | *CARD6* |  |  |  |  |
| *CELA1* | *HELZ, SERPINA1* | *CEP164,*  *GLI3, LPA,*  *PRRC2A* | *CCT2,*  *SIPA1L2* | *CELA1* | *PRRC2A,SERPINA1* |
| *CNTN4* | *PTPRG* | *PTPRF,* *PTPRG* | *PTPRD, PTPRS* |  | *CNTN4* |
| *CPNE1* | *CPNE3* |  |  |  | *CPNE3* |
| *CYP2A7* |  | *GNB2, SLC27A5* |  | *GNA11* | *GNB2* |
| *CYP4F8* |  |  | *DBNL* | *CYP4F12, , DBNL* |  |
| *DCANP1* |  | *DCANP1* |  |  |  |
| *DCHS2* | *DCHS1* | *DCHS2* |  | *DCHS1* |  |
| *DDX58* | *NLRC5, RNF135* | *CYLD,*  *PRKCB* | *MBP, PRKCG, SRC* | *PRKCA* | *MBP, NLRC5, PRKCG* |
| *DISC1* |  |  |  | *DISC1* | *DISC1* |
| *DMD* |  | *DMD* |  |  | *DMD* |
| *DNAH7* | *DNAH6* | *DNAH6* | *UBC* | *DNAH7* |  |
| *DOCK11* | *DOCK4* |  | *DOCK4, DOCK10* |  | *DOCK4* |
| *DOK3* | *DPP9* | *MIOS* |  |  |  |
| *ERBB2* |  | *ERBB2* |  |  | *ERBB2* |
| *FAM65C* |  |  |  | *FAM65C* |  |
| *FAM170A* |  | *USP7* | *VAPA, VAPB* |  | *VAPB* |
| *FDFT1* |  |  | *ANXA5* | *RUVBL1* | *ANXA5* |
| *FNBP4* | *ITSN2* | *ITSN1* | *ITSN1* | *ITSN2* | *ITSN2* |
| *GEMIN4* | *GEMIN5* | *GEMIN5* |  |  |  |
| *IL17RC* | *IL17RB* |  |  |  |  |
| *KANSL1* | *CTCF* | *DHX8, FOXK2, KRT15,* *RBBP5* | *DHX8, EXOC1, FOXK2, HSPB1, PHF20* | *KANSL2, ECI2, DISC1* | *CTCF, DISC1* |
| *KRT6C, KRT13/32/40* | *KRT7/20/80/84* |  |  | *KRT80* |  |
| *LILRB5* |  | *HLA-C* | *HLA-C* |  | *HLA-C* |
| *MAPK8IP1* | *MAPK8IP1* | *MAPK8* | *MAPK8IP1* |  |  |
| *MASP2* |  | *MASP2* |  |  |  |
| *MPRIP* |  |  | *MPRIP* |  |  |
| *MTUS1* |  | *SPICE1* | *CLTC, DCTN1* |  |  |
| *MUC16* | *MUC16* |  |  | *MUC16* |  |
| *NADK* | *INPP5E,*  *RASAL2,* *SRSF12, YWHAZ* | *GLRX3, INPP5E,*  *KIF13B,*  *MOV10,*  *ZNF638* | *AGAP1,CDK16,DCLK1, EEF1G,HDAC4,KRAS,*  *NAV1,SIPA1L1,TIAM1,*  *YWHAB/E/G/Q* | *NF1* | *YWHAB/E/G/Q/Z* |
| *NCOA3* |  | *CREBBP, EP300, ESR2, NCOR2* | *GSK3B, MAPK1, NCOA1, NCOA2* |  | *CREBBP, EP300, ESR2* |
| *NINL* |  |  |  | *TUBG1* |  |
| *NME4* |  |  | *NME1* |  |  |
| *OGFR* | *TRAP1* |  | *TRAP1* |  |  |
| *OR6C76* |  |  |  |  | *OR6C76* |
| *OR2T33, OR4B1, OR4K1/K2/K5/K15 OR4M1/N2/Q3, OR5B3, OR51B2* | *OR2M2,OR51M/V1, OR5P3, OR6B1/B3 OR52K2/N4,*  *OR11H4, OR13J1* | *OR4C46, OR11A1* |  | *OR2D2, OR5AC2, OR5M9, OR6N2, OR10Z1, OR11L1, OR51A2, OR52E6* | *OR6C1/C2/C3/C4/C6/C64P/C65/C68/70/C74, OR10A7/P1* |

| *PABPC4L* |  | *PABPC4L* |  |  |  |
| --- | --- | --- | --- | --- | --- |
| *PER3* |  |  | *PER1* | *PER1* | *PER3* |
| *PHC1* | *SCMH1* | *PHC2* | *CBX6, CBX8, PHC2,* | *CBX4,* *HDAC2* | *HDAC2* |
| *PKD1L2, PKD1L3* |  |  | *PKD1* |  |  |
| *PLEC* | *PLEC* |  | *PLEC* | *PLEC* | *PLEC* |
| *PLXNA3* | *PLXNA2* | *PLXNA1* | *PLXNA2, PLXNA4* | *PLXNB1* | *PLXNA2* |
| *PRSS48* | *PRSS38* | *PRSS38* |  |  |  |
| *PRSS55* | *PRSS38* | *PRSS38* |  | *PRSS55* |  |
| *PTCHD3* | *PTCHD3* |  |  |  |  |
| *RERE* |  |  | *RERE* |  | *RERE* |
| *RIOK2* | *G3BP1, TSR1 HNRNPUL1,* | *HECTD1, VPS16, VPS35* | *APP,* *HECTD1, RPS18, RPS25, VPS35* | *BEND3,* *EGFR,* *EWSR1,* *HECTD1,* | *EGFR, TSR1, VPS35* |
| *RBM23* | *ANKHD1* | *CLEC3A* | *GTF3C1, DBNL, TUBA4A* | *ANKRD17, DBNL, MAGED1, PDHA1* |  |
| *SGK223* | *SGK223* |  |  |  |  |
| *SIGLEC12* |  |  | *HSPA5, PTPN11* |  | *HSPA5* |
| *TSGA10IP* | *KRT20* | *KRT4, KRT20* |  |  |  |
| *ZNF45* |  | *CBX5* |  |  |  |
| *ZNF169* | *MRPS9,* *SOGA1,* *ZNF92* | *CLTCL1, SART3,SORL1* | *RPS3,* *SORL1* |  |  |
| *ZNF337* | *RPGRIP1* |  |  |  |  |
| *ZNF404* |  |  |  | *ZNF404* |  |
| *ZNF493* | *ACTL7B* | *ACTL7B* | *ACTL7B* | *ACTL7B* |  |
| *ZNF607* |  | *ZNF607* | *ZNF607* |  |  |
| **Genes from the 22q11.2 deleted region** | | | | | |
| *ARVCF* |  |  | *ARVCF* |  | *ARVCF* |
| *CDC45* | *MCM3, RFC1* | *CUL3* | *ACTR1A, PDS5B, TRAPPC3, UBC* | *BRCA2, L3MBTL1, MCM4,RPS6KB2* |  |
| *CLDN5* | *MPDZ* |  | *TJP1* | *MPDZ* | *CLDN5* |
| *CLTCL1* |  | *CLTCL1* |  |  |  |
| *COMT* | *CRBN, RGS2* | *CDC27, FN1, GBA* | *VCP* | *FN1, RAB2,*  *VCP* | *COMT* |
| *CRKL* |  | *CRKL* |  |  |  |
| *DGCR2** |  | *DGCR2* | *DGCR2* |  | *DGCR2* |
| *DGCR6** |  |  |  |  | *DGCR6* |
| *DGCR8** |  |  |  | *DGCR8* | *DGCR8* |
| *DGCR14* |  |  |  | *DGCR14* | *DGCR14* |
| *GNB1L* |  |  | *GNB1* |  | *GNB1L,GNB1* |
| *KLHL22* |  |  | *KLHL22* |  |  |
| *LZTR1* |  | *LZTR1* |  | *LZTR1* |  |
| *MED15* |  | *HIF1A, MED13/16 TRRAP* | *ATXN1,NCOA6,UBE2N MED13/14/16/29,* | *EWSR1, HIF1A, SMAD2, TRRAP* | *MED15* |
| *MRPL40* | *ATL3,* *KRTAP10-3 MRPL9, MRPL51,* *MRPS9, TSR1* | *USF2* | *ATP1A1,HADHA, HSPB1, KRTAP10-3 SCFD1* | *RBMS3* |  |
| *PI4KA* |  |  | *PI4KA* |  | *PI4KA* |
| *PRODH* | *CAPN11* | *HSPA8* | *HSPA2, HSPA6,* *TUBA4A* |  | *PRODH* |
| *RANBP1** | *G3BP1* | *MPG, SOAT1, UBR5* | *ATP6V1D, DLST, FN1, KPNB1, MYL6, RAB1A* | *EGFR, EWSR1, MDM2* | *RANBP1* |
| *RTN4R** |  |  | *RTN4R* |  | *RTN4R* |
| *SCARF2* |  |  |  | *SLC39A5* |  |
| *SEPT5** |  |  | *SEPT5* |  |  |
| *SLC25A1* |  | *SLC25A1* |  |  |  |
| *SNAP29* | *EXOC4* | *STX3* | *AP2A1, EHD1/D3,* *EXOC1/C4, NAPA* | *TSNARE1* |  |
| *TBX1* |  |  |  |  | *TBX1* |
| *TRMT2A** | *PBK, PIK3R3* | *DICER1, MOV10, PIK3R3* | *ANKRD17, APP, PIN1, ATP1B1, DICER1, MWD,EXOC3,HERC2* | *ATP1B1, DICER1, EGFR, FEZF2* | *TRMT2A* |
| *TXNRD2* |  |  | *EZR, NCOR1,NDUFA13* | *NCOA2/A6, NCOR1* |  |
| *ZDHHC8* | *CLDND1, ZDHHC5* | *ZDHHC5* |  |  | *ZDHHC8* |
| *UFD1L** | *FLNB, SUZ12, TAF1C* | *CHMP2A,DHX15,*  *HUWE1,ITPR1,MAP3K1MOV10, PDCD4, PSMD2,PTPRF,TAF1C* | *CFL1, HSPB1, LDHA, NSFL1C, STIP1, UBC, UBE2N, VCP* | *A2M, FAF2, ITPR1,MTMR12, VCP* | *UFD1L* |

Candidate genes that were identified for SZ sibling by all used models are presented. Genes overlapping with WES data are colored in purple. Genes that were reported in physical/pathway interaction with the identified candidate gene are colored in blue (GeneMania: <http://genemania.org/>; STRING: <http://string-db.org/>; NCBI/Gene/Interactors data base: https://www.ncbi.nlm.nih.gov/gene). *** Genes in 22q11.2 deleted region that were not covered by the current WES but were reported to be associated with SZ (NCBI database: <https://www.ncbi.nlm.nih.gov/gene/?term=schizophrenia>; VarElect: <http://varelect.genecards.org/>).

Supplementary Table 7 **DAVID functional candidate gene enrichments for the SZ phenotype**

|  | Function/Pathway accession | Function/Pathway description | P value | Gene count | Genes unique for SZ | Genes common to the three psychotic siblings | Genes common to SZ&DD or SZ&BPE |
| --- | --- | --- | --- | --- | --- | --- | --- |
| Signaling | GO:0004888 | Transmembrane  signaling receptor activity | 0.0000006* | 11 | *LILRB5,*  **OR genes** *OR4B1* | *ERBB2* | ***PLXNA3*** |
|  | KEGG:hsa04740 | Olfactory transduction | 0.00004* | 12 | **OR genes**, *OR51B2, OR4B1,* ***OR6C76*** |  | *OR2T33, OR5B3* |
|  | GO:0050906 | Detection of stimulus involved in sensory perception | 0.00008* | 13 | **OR genes**, *OR51B2, OR4B1,* ***OR6C76****, PKD1L3* |  | *OR2T33, OR5B3* |
|  | GO:0007600 | Sensory perception | 0.0007 | 16 | **OR genes**, *OR51B2, OR4B1,* ***OR6C76****, PKD1L3* | *COMT, TBX1* | *C2ORF71, OR2T33, OR5B3* |
|  | GO:0004930 | GPCR activity | 0.006 | 13 | ***OGFR****,* **OR genes**, *OR51B2, OR4B1,* ***OR6C76*** |  | *OR2T33, OR5B3* |
|  | GO:0007186 | GPCR signaling | 0.008 | 13 | **OR genes**, *OR51B2, OR4B1,* ***OR6C76*** | *GNB1L* | *OR2T33, OR5B3* |
| Neurodevelopment | GO:0030424 | Axon | 0.009 | 8 | ***MAPK8IP1*** | ***CNTN4****, SEPT5, CLDN5, RTN4R, RANBP1, COMT* | *DISC1* |
|  | GO:0050877 | Neurological system process | 0.01 | 18 | **OR genes**, *OR51B2, OR4B1,* ***OR6C76****, PKD1L3* | *CLDN5, COMT, DGCR2, TBX1* | *C2ORF71, OR2T33, OR5B3* |
|  | GO:0021602 | Cranial nerve morphogenesis | 0.01 | 3 |  | *TBX1* | *DMD,* ***PLXNA3*** |
|  | GO:0021545 | Cranial nerve development | 0.03 | 3 (1) |  | *DCNP1^1^, TBX1* | *DMD,* ***PLXNA3*** |
|  | GO:0048812 | Neuron projection morphogenesis | 0.047 | 8 | *CPNE1* | ***CNTN4****, ERBB2,* ***RERE****, RTN4R* | *DISC1, DMD,* ***PLXNA3*** |
|  | GO:1990138 | Neuron projection extension | 0.049 | 4 | *CPNE1* | *RTN4R* | *DISC1,* ***PLXNA3*** |
| Cytoskeleton activity | GO:0005856 | Cytoskeleton | 0.01 | 21 | ***KRT6C****,* ***DNAH7*** | *ACTN3, CDC45, CLDN5, CLTCL1, DGCR8, KLHL22, KRT13, KRT32, KRT40, MPRIP, RANBP1, SEPT5, SNAP29* | *DDX58, DMD, DISC1, MTUS1, NINL, PLEC* |
|  | GO:0007155 | Cell adhesion | 0.026 | 18 | ***DCHS2*** | *ACTN3, ARVCF, CLDN5,* ***CNTN4****, CRKL, ERBB2, DGCR2, DGCR6,* ***MPRIP****, PI4KA, RANBP1, SCARF2* | *DISC1, DMD, MUC16, PLEC, SIGLEC12* |
|  | GO:0044782 | Cilium organization | 0.049 | 5 | ***DNAH7*** | *C2ORF71, DISC1, DMD, SNAP29* |  |
| Lipid metha- bolism | GO:0046949 | [Fatty Acyl-CoA Biosynthesis](http://www.reactome.org/content/detail/75105) | 0.028 | 3 | ***ACOT8*** | ***ACSF2****, SLC25A1* |  |
|  | GO:0044255 | Cellular lipid metabolic process | 0.29 | 9 | ***ACOT8****, AMACR, CPNE1, CYP4F8,* ***FDFT1*** | ***ACSF2****, ERBB2, PI4KA* | *CYP2A7* |
| Histone  modification | GO:0006325 | Chromatin organization | 0.69 | 5 | ***KANSL1****,* ***NCOA3*** | *CDC45,* ***PHC1****,* ***RERE*** |  |
|  | GO:0016570 | Histone modification | 0.75 | 3 (3) | ***KANSL1****,* ***NCOA3*** | *CLTCL1* ^2^, ***CNTN4*** ^3^, ***PHC1****,* ***RERE*** |  |
|  | UP_SEQ_  FEATURE | Charge relay system | 0.02 | 5 | ***ACOT8****,* ***CELA1****, PRSS48,* ***PRSS55*** |  | *MASP2* |

The analysis included all bioinformatically selected genes for SZ phenotype (Supplementary Table S6.) ^*^ Represents p-values, remained significant after multipule testing (Benjamini correction p<0.05). Genes including rare (<0.05), *de-novo* and CNV variants, excluding 22q11.2-loss, are bolded. OR genes – seven olfactory receptor genes clustered on 14q11.2-gain (*OR4K1, OR4K2, OR4K5, OR4K15, OR4M1, OR4N2*, and *OR4Q3*). GPCR- G-protein coupled receptor. Underlined genes represent additional to DAVID results annotations, including NCBI –BioSystem data base (<https://www.ncbi.nlm.nih.gov/biosystems>) and relevant publications:

^1^ Schröder, J.C. *et al*. A boy with homozygous microdeletion of NEUROG1 presents with a congenital cranial dysinnervation disorder [Moebius syndrome variant]. *Behav Brain Funct.* **9**, 7 (2013). ^2^ Weinstein, J.S. *et al.* Global transcriptome analysis and enhancer landscape of human primary T follicular helper and T effector lymphocytes. *Blood* **124**, 3719-29 (2014). ^3^ Shulha, H.P*. et al*. Human-specific histone methylation signatures at transcription start sites in prefrontal neurons. *PLoS Biol*.**10**, 11 (2012).
